# Impact of Relative Change in Temperature and Atmospheric Pressure on Acute Aortic Syndrome Occurrence in France

**DOI:** 10.1038/s41598-019-56841-w

**Published:** 2020-01-09

**Authors:** Guillaume Guimbretière, Simon Nusinovici, Antoine Monnot, Jonathan Sobocinski, Thomas Sénage, Pascal Delsart, Pierre-Antoine Gourraud, Blandine Maurel

**Affiliations:** 1 Department of Vascular Surgery, Institut du Thorax, CHU Nantes, Nantes, France; 2 Cellule d’Epidémiologie Clinique/Clinique des données, CIC de Nantes, Nantes, France; 3 Aortic Centre, CHRU Lille, Lille, France; 4 Department of Cardiothoracic Surgery, Institut du Thorax, CHU Nantes, Nantes, France

**Keywords:** Health care, Risk factors, Signs and symptoms

## Abstract

Acute aortic syndromes (AAS) have been related to significant circadian and seasonal conditions. We used time series analyses to study the impact of meteorological variations on AAS occurrence. We retrospectively assessed 140 patients presenting with AAS over a 6-year period in a French university hospital. Average daily temperature (T) and atmospheric pressure (AP) at the location of the event were collected within the previous 10 days, and their association with AAS investigated with generalized additive models. A decrease in temperature of more than 5 °C within the previous seven days was significantly associated with an increased risk of AAS occurrence (OR equal to 1.86 [1.06; 3.44]). Subgroup analysis revealed that the risk was only significant among normotensive individuals (n = 41) free from blood pressure lowering medication (OR equal to 2.3 [1.05; 5.37]), but not among hypertensive individuals under blood pressure lowering medication despite a larger patient number (n = 99). Similarly, only among the subgroup of normotensive individuals a decrease of AP between 2 and 4 kPa within the previous 3 days was associated with an increased risk of AAS (OR equal to 2.93 [1.1; 8.15]) and an increased between 2 and 4 kPa was associated with a decreased risk (OR equal to 0.59 [0.36; 1.00]). Variations of meteorological conditions (temperature and AP) within the previous week seem to have effects on triggering AAS especially among the population free from blood pressure lowering medication.

## Introduction

Similar to other cardiovascular adverse events, acute aortic syndromes (AAS) exhibit significant circadian and seasonal variations. Characterizing and understanding these variations is essential to ensure an optimal management of medical resources throughout the year and treatment strategy during vulnerable periods. Several studies have investigated the influence of climatic conditions on AAS^1–6^, reporting a significant occurrence of AAS in the morning and a correlation with low temperature and winter season^2–5^. However, most of these studies are descriptive or investigate the impact of daily temperature only^5^, even if climatic variation throughout several consecutive days may have also been of major importance^3^.

Time series analyses are widely used to model the influence of climatic factors on health outcomes^7,8^. These models allow for the modeling of the impact of delayed effects of climatic factor variations, while accounting for temporal processes and adjusting for potential confounding factors. The impact of variations in climatic conditions may depend on local context such as the initial value at which the variation starts. A decrease in temperature starting from a high temperature could indeed have a different effect compared to a decrease from a low value. Therefore, rather than absolute climatic conditions, their relative changes may be of interest^9^. To our knowledge, these models have not been used in acute aortic syndromes.

The objective of this study was to investigate the effects of both relative and absolute change in temperature and atmospheric pressure on the risk of AAS. Specifically, we assessed the possible delayed effect of these climatic factors within 10 days prior to the event; the effect of the initial values of climatic conditions; and the patterns of relationships between climatic conditions and the risk of AAS.

## Methods

This study was a retrospective, monocentre observational trial in a university hospital located in Nantes (France), and has been approved by the local research ethics committee the GNEDS (Groupe Nantais d’Ethique dans le Domaine de la Santé) and the CNIL (Commission Nationale de l’Informatique et des Libertés); the study reference number was RC17_0181. Participation required informed consent obtain by phone and compliance with the study inclusion and exclusion criteria. We combined hospital records-based information with local weather forecast archives. All methods were performed in accordance with the relevant guidelines and regulations.

### Population

All consecutive patients admitted for AAS from January 2007 to December 2016 were considered for inclusion in the study. AAS included Stanford type A acute aortic dissections (AAD) or intramural hematoma and Stanford type B AAD or intramural hematoma. The diagnosis of AAS was based on computed tomography angiography. Patients living beyond a 100 km radius from a meteorological station were excluded to avoid information bias due to inaccurate data.

Type A AAD underwent surgery in all cases with subsequent histopathological examination of the aortic wall; type B AAD underwent either best medical treatment or endovascular repair if complicated. Case collected characteristics were: type of AAS, age, gender, body mass index, cardiovascular risk factors, date and location at the time of AAS occurrence. Data were collected from the hospital registry, and included every patient who arrived alive at the hospital (patients who succumbed to the AAS event before reaching the hospital were not included in this study).

### Climatic data

Nantes is located in the west part of France and has a temperate oceanic climate. It presents with an urban, suburban and country catchments area of about 2 900 000 inhabitants. Climatic data were obtained from Météo-France web service, the national meteorological service of France, which compiles raw data from Nantes meteorological station various sources and re-analyses the data using weather prediction models. Data consisted of temperature and atmospheric pressure collected daily every 3 hours over the past 10 years. Average daily temperatures and atmospheric pressures were calculated for each area and then combined with clinical data. This approach assumed that in a specified geographic area all persons experience the same exposure.

### Statistical analyses

The associations between climatic factors and the risk of AAS were investigated with generalized additive models. Due to the sparse distribution of cases over days (a very low number of days with more than one case), daily occurrence of AAS was considered as a binary outcome. Long-term trends and seasonality were filtered out in order to account for potential confounding factors, such as changes in health status over time or atmospheric pollution, while retaining uncompounded shorter-term fluctuations possibly associated with short-term variations of climatic factors.

Prior to the study of short-term climatic factors per se, a regional model was set to remove long-term trend and seasonality. Six degrees of freedom (6 years of inclusion) were chosen for the annual long-term trends in the areas of Nantes to capture bi-monthly variations of occurrence of AAS. The analyses required two stages. The first stage consisted in the identification of a possible delayed effect of the variations of climatic factors on the risk of AAS (identification of time lags). Day 0 was the day of occurrence of the AAS, and day -n was the day “n” days prior to the AAS. The description of the statistical modeling strategy is detailed in the supplemental material. Briefly, automatic term selections were used to identify the time lags for temperature and atmospheric pressure associated with variations of risk of AAS. Four degrees of freedom were used for each climatic variable to account for non-linearity.

In the second step, the effect of variations in temperature and atmospheric pressure on the risk of AAS - according to the initial value at which the variation starts (absolute vs. relative effect) - was investigated. Therefore, for each time lag of temperature and atmospheric pressure identified in the first step, two possible variables were considered: the variation between day 0 and day -n alone or its interaction with the initial value at day -n. Then, for each time lag considered, the different models were compared using Akaike’s Information Criteria (AIC). All models are presented in the Supplementary Materials section. Finally, subgroup analyses we performed according to the hypertensive status and the type of AAS. We defined as “normotensive patient” every patient without previous medical history of hypertension and free from blood pressure lowering medication; and as “hypertensive patient” every patient under blood pressure lowering medication at the time of the event. P-values < 0.05 were considered to be statistically significant. Statistical analyses were performed using R software (version 3.5.1).

## Results

**Figure 1:**
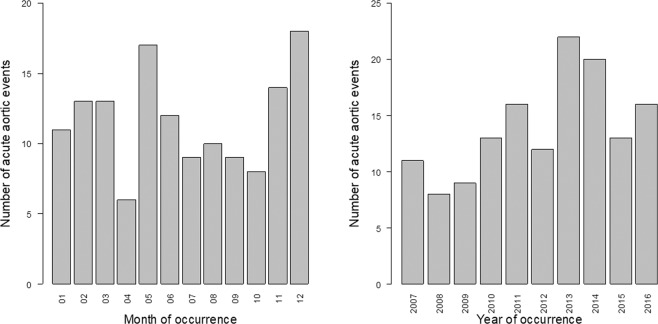
Number of cases of acute aortic syndrome per year and per month (n = 140).

A total of 140 patients living within the studied area have been admitted with AAS diagnosis over the 6-year studied period. Eleven patients were excluded because living beyond a 100 km radius from the meteorological station. The cumulative daily incidence of AAS (number of days with at least one case / total number of days during the study period) was 0.038 with 138 days with one or more patients admitted for AAS. The number of AAS in both areas showed variations within months and years (Fig. 1), with significantly more cases in May (P = 0.03) and December (P = 0.03) compared to April, and in 2013 (P = 0.01) and 2014 (P = 0.04) compared to 2008. Patients’ characteristics are described in Table 1.

**Table 1:** Patients’ characteristics.

	Nantes
	(N = 140)
**Gender**	
F	33 (23.6%)
M	107 (76.4%)
**Age**	
Meam (SD)	62.1 (12.4)
**BMI (kg.m**^**2**^**)**	
Mean (SD)	27.5 (5.1)
**Cardiovascular risk factors**	
HBP	99 (70.7%)
DM	8 (5.7%)
Tabaco use	63 (45.0%)
Dyslipidemia	29 (20.7%)
Histological alteration of the media (among type A AAD)	51 (42.5%)
**AAS**	
Type A AAD	120 (85.7%)
Type B AAD	20 (14.3%)

BMI: body mass index; IQR: interquartile range; HBP: high blood pressure; DM: diabetes mellitus; AAD: acute aortic dissection.

**Figure 2:**
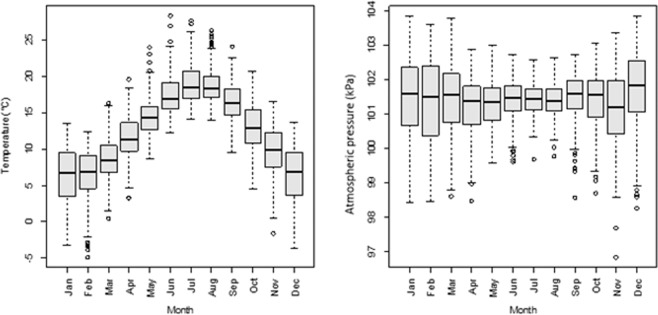
Variations of average temperature and atmospheric pressure within each month (between 2007 and 2016).

The monthly average of temperatures ranged from 4.1 to 18.4 °C, and the monthly average of atmospheric pressures from 100.9 to 101.3 kPa (Fig. 2). There was a significant higher temperature between march and October (all P < 0.001) compared to January, and a higher atmospheric pressure in April (P = 0.001), May (P = 0.04), November (P < 0.001) and December (P = 0.001) compared to January. The variations of climatic conditions, modeled using long-term trend and seasonality components, are presented in the Supplemental Material Section (S1).

### Delayed effect of climatic conditions and impact of initial values on AAS

All the intermediate results are presented in the Supplemental Material S1 to S4.

### Effect of temperature change on AAS

**Figure 3:**
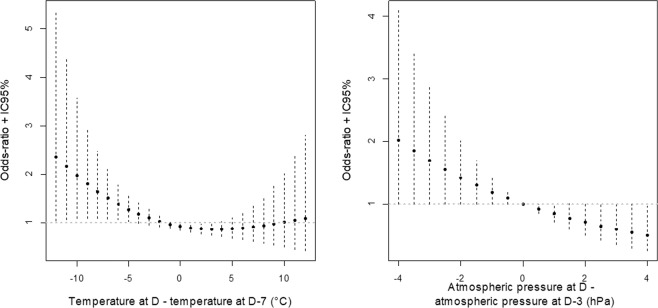
Variations of risk of acute aortic syndrome, expressed as Odds-Ratio (OR) with their 95% Confidence Intervals (CI), associated with (**A**) the difference between temperature at day 0 and day -7; and (**B**) the difference between atmospheric pressure at day 0 and day −3 (model 2 with Npa = 3 in Table 1 Supplemental Material).

The difference in temperatures between day 0 and day −7 was associated with a significant increased risk of AAS occurrence at day 0. A decrease in temperature (more than 5 °C) within the previous seven days was associated with an increased risk of AAS (average OR equal to 1.86 [1.06; 3.44]) (Fig. 3, Table 2). Subgroup analysis revealed that the risk was only significantly increased among “normotensive patients” (average OR equal to 2.3 [1.05; 5.37]) despite a limited number of patients (n = 41) (among “hypertensive patient” OR was equal to 1.33 [0.79; 2.31]).

**Table 2:** Risk of acute aortic syndrome (AAS) according to the temperatures.

Variation in T between day −7 and day 0^§^	Total OR [95% CI] (n = 138)^§§^	Normotensive OR [95% CI] (n = 41)	Hypertensive OR [95% CI] (n = 99)	Type A AAD OR [95% CI] (n = 120)
[−13 °C; −5 °C]	1.86 [1.06; 3.44]	2.3 [1.05; 5.37]	1.33 [0.79; 2.31]	1.96 [1.14; 3.51]
]−5 °C; 5 °C [	0.97 [0.84; 1.11]	1 [0.8; 1.25]	0.99 [0.86; 1.14]	0.97 [0.83; 1.13]
[5 °C; 13 °C]	0.99 [0.54; 1.96]	0.6 [0.26; 1.54]	0.91 [0.54; 1.6]	0.94 [0.51; 1.88]

OR: Odds-Ratio; CI: Confidence Intervals; T: temperatures; normotensive: patient free from blood pressure lowering medication; hypertensive: patient under blood pressure lowering medication at the time of the event; type A AAD: patients presenting with type A acute aortic dissection.

^§^A negative value corresponds to a decrease of temperature during the previous 7 days; ^§§^n = number of days with at least one AAS.

### Effect of atmospheric pressure change on AAS

An increase in atmospheric pressure (between 2 and 4 kPa within the previous 3 days) was associated with a decreased risk of AAS (average OR equal to 0.59 [0.36; 1.00]) (Fig. 3, Table 3). Similarly to temperature analysis, subgroup analysis revealed that the impact of atmospheric pressure on AAS was significant among the subgroup of “normotensive patients” but not among the subgroup of “hypertensive patients”. Among “normotensive patients”, an increase in AP was significantly associated with a reduced risk of AAS (OR equal to 0.36 [0.15; 0.91]) and a decrease in AP was significantly associated with an increased risk (OR equal to 2.93 [1.1; 8.15]), (Fig. 3, Table 3).

**Table 3:** Risk of acute aortic syndrome according to the atmospheric pressures.

Variation in AP between day −3 and day 0^§^	Total OR [95% CI] (n = 138) ^§§^	Normotensive OR [95% CI] (n = 41)	Hypertensive OR [95% CI] (n = 99)	Type A AAD OR [95% CI] (n = 120)
[−4 kPa; −2 kPa]	1.70 [0.99; 2.97]	2.93 [1.1; 8.15]	1.42 [0.77; 2.69]	1.3 [0.73; 2.36]
]−2 kPa; 2 kPa [	1.01 [0.87; 1.19]	1.06 [0.82; 1.4]	1.01 [0.85; 1.21]	1 [0.85; 1.19]
[2 kPa; 4 kPa]	0.59 [0.36; 1.00]	0.36 [0.15; 0.91]	0.71 [0.39; 1.31]	0.77 [0.439; 1.37]

OR: Odds-Ratio; CI: Confidence Intervals; AP: atmospheric pressures; ^§^a negative value corresponds to a decrease of atmospheric pressure; ^§§^n = number of days with at least one AAS; normotensive: patient free from blood pressure lowering medication; hypertensive: patient under blood pressure lowering medication at the time of the event; type A AAD: patients presenting with type A acute aortic dissection.

## Discussion

This study reports a risk of AAS triggering event related to both variation in temperature and atmospheric pressure within the last week among a population of patient free from arterial blood pressure medication. Indeed, although considerable advances in identifying the conditions that may predispose to AAS have occurred, little is known about the incident event leading to the acute event. In 2002, Mehta *et al*. analyzed patients enrolled in the International Registry of Acute Aortic Dissection (IRAD), and they reported for the first time in large scale a high circadian variation and a frequency of AAS significantly higher during winter with a peak in January^2^. From this report, many studies^1–6^ had worked on this topic and exhibited significant circadian and seasonal monthly variations but only one reported statistically significant differences between decrease in outdoor temperature and incidence of AAS. In 2010, Benouaich *et al*. raised the key role of temperature decreasing during the day before AAS and suggested an interesting way that relative change in temperature could be a triggering factor rather than the absolute temperature^3^.

In the French area considered in this study, variations of temperature and atmospheric pressure were found to be associated to the risk of AAS within the following days. We reported that a decrease in temperature within the seven previous days was associated with an increased risk of AAS. Regarding the variations of atmospheric pressure, an increase within the previous three days was associated with reduced risk of AAS. These results suggest, as also reported for rupture of abdominal aortic aneurysms^10,11^ and acute myocardial infarction^12^, a complex relationship between seasons and pathophysiological exogenous and endogenous individual factors.

Mechanisms leading to the possible influence of external weather on AAS onset are most likely multifactorial. It has been confirmed that blood pressure variations influence the incidence of AAS, and there are also correlations between hypertension and atmospheric temperature. Bai *et al*. reported that compared to the temperature with minimum risk of morbidity, cold temperatures were associated with a 37% increase in hypertension-related hospitalizations whereas no significant association with hot temperatures (99^th^ percentile) was observed^13^. In a large population-based analysis, Alpérovitch *et al*. reported that blood pressure was strongly influenced by outdoor temperature especially in the elderly, with a systolic blood pressure increase of 0.8 mm Hg in subjects aged 65 to 74 years for a 15 °C decrease in outdoor temperature^14^. The acute cold-induced rise in blood pressure is caused by the activation of the autonomic nervous system where the cooling-related sympathetic response results in vasoconstriction and subsequently increased blood pressure and higher cardiac workload^15^.

The only physiological parameter associated with atmospheric pressure is arterial blood gas concentration^16^, even if the exact mechanism by which reduced arterial oxygen tension may trigger an aneurysm rupture or a dissection remains unknown. A hypothesis is that changes in atmospheric pressure may increase transmural arterial stress by transiently lowering tissue pressure in respect to blood pressure creating a net expansive force on a weakened aneurysmal wall prone to rupture. Additionally, atmospheric pressure may also increase blood pressure and consequently the shear stress across the weakened aneurysm wall^17^.

Interestingly in our study, subgroup analysis revealed that external weather impacted only patients free from blood pressure lowering medication at the time of the event. These results suggest these medications, reducing endothelial dysfunction, may decrease the impact of external factors on the arterial wall shear stress.

In addition to the well described winter peak in AAS, a second peak in April/May was observed in our study. In 2003, McCarthy *et al*.^10^ from the university of Leicester were the first to highlight this second peak of AAS and raised the hypothesis that their finding was due to either a local geographical factor or a result of the smaller sample size (n = 223). Our study suggests that another explanation may be the high variation of temperature and atmospheric pressure during these months.

This varied impact of climatic factors suggests the existence of multiple determinants influencing the risk of AAS occurrence. Edwin *et al*.^18^ exposed that pathogenesis of AAS could be the result of the interaction of three factors: (i) a predisposition provided by an abnormality or weakening of the aortic media, (ii) any agent of intimal injury or tear resulting in the intima medial flap, and (iii) hemodynamic factors that propagate the dissection once it has been initiated. Clearly, climatic conditions only represent a precipitating factor for AAS, in addition to predisposing factors such as media histological alterations for dissections. In the subgroup analysis of patients with histopathological alteration of the media, we failed to demonstrate a significant variation in the impact of the climatic conditions as compared with other patients, possibly because of a lack of statistical power.

Strength of this study was the inclusion of cases which covered quite long time periods (6 years). Appropriate statistical models were used to investigate the effects of both temperature and atmospheric pressure while removing the effects of seasonality and long-term trends. The results involving interactions between the changes in climatic factors and their initial values confirm the interest of considering such models that allow modeling complex and non-linear relationships.

A limitation of this study was its ecological design, i.e. climatic data were not individually collected but were attributed to individuals that lived near to the meteorological station. The risk is, therefore, to attribute data to individuals that would not be representative of their close environments. In order to limit this risk, only cases living close to the meteorological stations were included (within 100 km). This selection consequently limited the number of cases, with a number of cases quite limited for time series models involving complex term interactions. Another limitation of our study was that the role of other weather variables, like relative humidity or the integrative body heat index, were not included in the analysis. Moreover, due to the retrospective design of the study, it was not possible to include in the analysis some parameters like the control of a hypertension as well as its severity and their correlation with the event, or the patient’s medication. Finally, patients who died from their condition prior to hospital admission were not included in this work.

We report here that short term changes in temperature or atmospheric pressure have a significant impact on AAS occurrence, depending on an upward or downward variation. Subgroup analysis revealed that external factors had an impact only in patients free from blood pressure lowering medication. The use of appropriate models allowed us to demonstrate these complex relationships, accounting for possible confounding factors, temporal correlation of the data, identifying lag time effect and modeling non-linear relationships. These finding may help to tailor preventive strategies for high risk patients and to anticipate a higher influx of AAS to optimize surgical and medical resources throughout the year. Future studies with larger scale are required to assess this statistical model and confirm our promising approach.

## Supplementary information

dataset 1.
