# Supplementary material for: Impact of Relative Change in Temperature and Atmospheric Pressure on Acute Aortic Syndrome Occurrence in France
Source: Sci Rep. 2020 Jan 9;10:76. doi: 10.1038/s41598-019-56841-w (PMC6952440; doi:10.1038/s41598-019-56841-w)
Supplement: Supplementary file 1 — dataset 1. [file 41598_2019_56841_MOESM1_ESM.docx]

# IMPACT OF RELATIVE CHANGE IN TEMPERATURE AND ATMOSPHERIC PRESSURE ON ACUTE AORTIC SYNDROME OCCURRENCE IN FRANCE

Guillaume Guimbretière, MD^1^ and Simon Nusinovici, PhD^2^, Antoine Monnot, MD^3^, Jonathan Sobocinski MD, PhD^3^, Thomas Sénage MD, PhD^4^, Pascal Delsart MD^3^, Pierre-Antoine Gourraud, MD, PhD^2^, Blandine Maurel, MD, PhD^1^

^1^Department of Vascular Surgery, Institut du Thorax, CHU Nantes

^2^Cellule d'Epidémiologie Clinique / Clinique des données - CIC de Nantes

^3^ Aortic Centre, CHRU Lille

^4^ Department of Cardiothoracic Surgery, Institut du Thorax, CHU Nantes

**SUPPLEMENTAL MATERIAL**

S1. Identification of delayed effect (time lags) of the variations of climatic factors on the risk of acute aortic events. Two different models were considered for the temperature and the atmospheric pressure. Automatic term selection was considered identify the time lag(s) associated with variations of the risk of acute aortic events. Long-term trend and seasonality were considered in all the models.

|  | P-value |
| --- | --- |
| Temperature at day 0 - temperature at day -1 | 0.85 |
| Temperature at day 0 - temperature at day -2 | 0.71 |
| Temperature at day 0 - temperature at day -3 | 0.53 |
| Temperature at day 0- temperature at day -4 | 0.49 |
| Temperature at day 0- temperature at day -5 | 0.41 |
| Temperature at day 0- temperature at day -6 | 0.79 |
| Temperature at day 0- temperature at day -7 | 0.02 |
| Temperature at day 0- temperature at day -8 | 0.46 |
| Temperature at day 0- temperature at day -9 | 1.00 |
| Temperature at day 0- temperature at day -10 | 1.00 |
| Atmospheric pressure at day 0 - atmospheric pressure at day -1 | 0.51 |
| Atmospheric pressure at day 0 - atmospheric pressure at day -2 | 0.43 |
| Atmospheric pressure at day 0 - atmospheric pressure at day -3 | 0.04 |
| Atmospheric pressure at day 0 - atmospheric pressure at day -4 | 1.00 |
| Atmospheric pressure at day 0 - atmospheric pressure at day -5 | 1.00 |
| Atmospheric pressure at day 0 - atmospheric pressure at day -6 | 0.77 |
| Atmospheric pressure at day 0 - atmospheric pressure at day -7 | 0.92 |
| Atmospheric pressure at day 0 - atmospheric pressure at day -8 | 0.58 |
| Atmospheric pressure at day 0 - atmospheric pressure at day -9 | 0.65 |
| Atmospheric pressure at day 0 - atmospheric pressure at day -10 | 0.52 |

S2. Model selection using Akaike's Information Criteria (AIC).

|  | Temporal variables | Variables regarding T | Variables regarding AP | Npa=3 |
| --- | --- | --- | --- | --- |
| Model 1 | Long-term trend and seasonality | T at D0 - T at D-7 | AP at D0 - AP at D-Npa | **1134.6** |
| Model 2 | Long-term trend and seasonality | Interaction (T at D0 - T at D-7) x (average T at D-7) | AP at D0 - AP at D-Npa | 1136.5 |
| Model 3 | Long-term trend and seasonality | T at D0 - T at D-7 | Interaction (AP at D0 - AP at D-Npa) x (average AP at D-Npa) | 1137.1 |
| Model 4 | Long-term trend and seasonality | Interaction (T at D0 - T at D-7) x (average T at D-7) | Interaction (AP at D0 - AP at D-Npa) x (average AP at D-Npa) | 1139.2 |

*Npa: Number of days considered for the time lag (for example, Npa = 3 corresponded to the difference of atmospheric pressure between day 0 and day -3);D: day; T: temperature; AP: atmospheric pressure*

S3. Variables significance of models with the lowest Akaike's Information Criteria (AIC) identified in Supplemental Material Table 1.

|  | model 2 (Npa=3) |
| --- | --- |
|  | P-value |
| Long-term trend | 0.05 |
| Seasonality | 0.43 |
| Variables regarding temperature | 0.06 |
| Variables regarding atmospheric pressure | 0.05 |

*Npa: Number of days considered for the time lag (for example, Npa = 3 corresponded to the difference of atmospheric pressure between day 0 and day -3).*

S4. Variations of risk of acute aortic events, expressed as Odds-Ratio (OR) with their 95% Confidence Intervals (CI), associated with long-term trend (A) and (B) seasonality. No climatic factors were considered in these models.
